# Evaluating the safety, pharmacokinetics and efficacy of phage therapy in treating fracture-related infections with multidrug-resistant *Staphylococcus aureus*: intravenous versus local application in sheep

**DOI:** 10.3389/fcimb.2025.1547250

**Published:** 2025-04-04

**Authors:** Christian Peez, Baixing Chen, Leopold Henssler, Marco Chittò, Jolien Onsea, Michael H. J. Verhofstad, Daniel Arens, Caroline Constant, Stephan Zeiter, William Obremskey, Andrej Trampuz, Michael J. Raschke, Charalampos Zalavras, Willem-Jan Metsemakers, T. Fintan Moriarty

**Affiliations:** ^1^ Department of Trauma, Hand and Reconstructive Surgery, University Hospital Münster, Münster, Germany; ^2^ AO Research Institute Davos, Davos, Switzerland; ^3^ Department of Trauma Surgery, University Hospitals Leuven, Leuven, Belgium; ^4^ Department of Development and Regeneration, KU Leuven, Leuven, Belgium; ^5^ Department of Trauma Surgery, University Hospital Regensburg, Regensburg, Germany; ^6^ Trauma Research Unit, Department of Surgery, Erasmus MC, University Medical Center Rotterdam, Rotterdam, Netherlands; ^7^ Center for Musculoskeletal Research, Vanderbilt University Medical Center, Nashville, TN, United States; ^8^ Queensland University of Technology, Brisbane, QLD, Australia; ^9^ Department of Trauma, Hand and Reconstructive Surgery, University Hospital Muenster, Muenster, Germany; ^10^ Department of Orthopaedic Surgery, Los Angeles General Medical Center & Keck School of Medicine, University of Southern California, Los Angeles, CA, United States

**Keywords:** bacteriophage, fracture related infection, MRSA, pharmacokinetics, administration, neutralization, osteomyelitis, *Staphylococcus aureus*

## Abstract

**Background:**

Fracture-related infections (FRI), particularly those caused by antibiotic resistant *Staphylococcus aureus*, present significant clinical challenges due to the formation of biofilm on the implanted device, and reduced options for conventional antibiotic treatment. Bacteriophage (phage) therapy (PT) offers a targeted approach to managing such infections, however, evidence for pharmacokinetics and optimal route of administration is limited for FRI. This study aimed to evaluate safety, phage distribution kinetics, phage neutralization, and antibacterial efficacy after intravenous or local administration in a sheep model.

**Methods:**

The study was conducted in two phases: Phase 1 assessed the safety and distribution of two successive rounds of intravenous and local phage administration in non-infected sheep, while Phase 2 evaluated the therapeutic efficacy of intravenous versus local phage administration in combination with intravenous vancomycin in treating MRSA-induced FRI (tibial osteotomy with plate fixation). The specific pathogen and phage used in the sheep were both taken from a human FRI patient treated with PT. Phage neutralization and phage distribution were the primary outcomes measured in both phases of the sheep study.

**Results:**

Both intravenous and local phage administration were well-tolerated in non-infected sheep. Phages were cleared rapidly from circulation after intravenous administration, with no phage detected after 240 minutes. Phage neutralization increased during PT, peaking at 99.9% in non-inoculated sheep by the end of the second phage treatment (day 50). In infected sheep, phage neutralization levels reached a maximum of 99.9% earlier (day 13), with no significant differences between intravenous and local administration. The bacterial load was not significantly changed by PT, either IV or locally applied.

**Conclusions:**

PT is a safe adjunct to antibiotic treatment for FRI, however, phage neutralization developed rapidly and was accelerated in infected hosts. Further research is required to optimize phage selection, dosing, and delivery methods to enhance its therapeutic potential as an adjunct to conventional antibiotic therapy, particularly in the face of challenges such as rapid clearance and phage neutralization.

## 1 Introduction

Fracture-related infection (FRI) represents a significant clinical challenge, particularly in the presence of antibiotic-resistant bacteria and biofilms, leading to prolonged hospital stays, repeated surgeries, high recurrence rates and, in severe cases, amputation or even death (Moriarty et al., 2022; Metsemakers et al., 2023a). Methicillin-resistant *Staphylococcus aureus* (MRSA) is an important causative pathogen in these infections, further complicating treatment and worsening patient outcomes (Morgenstern et al., 2021). Central to the treatment of FRI are biofilms, which are complex communities of bacteria that adhere to surfaces and are encased in a self-produced protective extracellular matrix (Khatoon et al., 2018). Growth in biofilm helps bacteria evade immune defenses and exhibit tolerance to antibiotics (Masters et al., 2019). Standard procedures for managing FRI involve surgical debridement, irrigation, implant removal, and prolonged antibiotic administration (Metsemakers et al., 2020). Despite these extensive efforts, the recurrence rates remain high, necessitating the exploration of alternative and adjunctive therapies (Lebeaux et al., 2014).

Bacteriophages, or phages, are viruses that specifically infect and lyse bacterial cells, offering a targeted approach to eradicating infections (Onsea et al., 2020). Recent case reports and small-scale clinical studies have shown encouraging results for phage therapy (PT), including for antibiotic-resistant infections and orthopaedic device-related infection (Uyttebroek et al., 2022). A review of 137 human patients who received phage therapy for orthopedic infections reported that direct local instillation was the most commonly used method (35.0%). This is followed by combined intravenous and intra-articular delivery (16.1%), combined intra-articular and direct local delivery (16.1%), and intravenous delivery alone (15.3%) (Young et al., 2024). While local administration ensures high concentrations of phages directly at the infection site, this approach increases the risk of superinfection when percutaneous drains are left *in situ* for multiple days to facilitate repeat dosing (Onsea et al., 2019). In contrast, local administration may delay some of the potential side effects associated with IV administration, such as immune-mediated neutralization (Suh et al., 2022; Le et al., 2023).

The titer of phage reaching infected musculoskeletal tissues is poorly understood for both modes of administration. There is also uncertainty on how antibacterial efficacy compares between local and IV administration of PT. To address these knowledge gaps, we performed a series of PT investigations in a large animal model of FRI. The phage and pathogen (more information can be found in the Methods section) were selected from a successful PT case in a human patient (Onsea et al., 2021b) and transferred to a large animal model to provide the best possible recapitulation of the key factors involved in PT in FRI: size of the animal, volume of phage applied, and use human implants to fix an osteotomy. The large animal study was performed in two phases, both of which compared IV with local administration: Phase 1 focusing on safety and distribution in non-infected sheep, and Phase 2 focusing on therapeutic efficacy in sheep with established MRSA FRI. Primary outcome measures were phage distribution and phage neutralization, with secondary outcome being antibacterial efficacy in phase 2. The overall goal is to provide more evidence and data for PT in FRI, contributing to more effective and safer PT protocols for clinical use.

## 2 Methods

### 2.1 Bacterial strains and inoculum preparation

This patient from whom the pathogen was cultured was included in the PHAGEFORCE study (NCT06368388) which aims to standardize PT and prospectively collect data on patients (Onsea et al., 2021b). In brief, the patient suffered from an FRI after a Gustilo-Anderson type II open tibia fracture. Despite multiple surgical revision procedures and multiple courses of antibiotic treatment, the infection persisted. Deep tissue cultures showed the presence of MRSA (MRSA MSI-003), resistant to oxacillin, levofloxacin, gentamicin, tobramycin, erythromycin, clindamycin, minocycline, and rifampicin. The Coordination group for Bacteriophage therapy Leuven (CBL) considered previous surgical and antibiotic treatments as adequate and maximal, thereby deeming the patient eligible for PT. During a surgical debridement, a draining system was placed and based on susceptibility testing, the patient received 20 mL of single phage ISP (10^7^ PFU/mL) three times per day for 10 days postoperatively. The patient simultaneously received antibiotic treatment for a total duration of three months. No adverse events were reported during PT. After cessation of treatment and over three years in follow-up, the infection did not recur.

Given the successful clinical outcome observed in the patient treated with single phage ISP, this study aimed to further investigate the safety, pharmacokinetics and efficacy of PT using a large animal model with the same pathogen and phage. MRSA MSI-003 was kept at -20°C in a 25% glycerol solution for long-term storage. Bacteria were grown on tryptic soy agar (TSA, Sigma-Aldrich, Steinheim, Germany) and an overnight culture of MRSA MSI-003 was prepared in 20 ml tryptic soy broth (TSB, Sigma-Aldrich, Steinheim, Germany) at 37°C one day before animal surgery. A fresh logarithmic phase culture was prepared in TSB from the overnight culture. Bacteria were centrifuged at 3220 × g for 10 min (Thermo fisher) and washed twice in phosphate buffered saline (PBS, Sigma-Aldrich, St. Louis, MO, USA) 1.5h prior to surgery and diluted in PBS to obtain a target inoculum of 2.0 × 10^6^ CFU/mL. A total of 1mL of this suspension was used to inoculate the osteotomy site and induce infection. The bacteria were applied within 30 minutes of the inoculum preparation. Immediately after inoculum preparation, the number of bacteria present in each inoculum was quantified by plating a dilution series into TSB at 37°C.

### 2.2 Overview of animal study

Institutional review board approval for the animal study was granted by the Ethical Committee of the Canton of Grisons in Switzerland (approval number: GR 10_2023). The experiments were conducted in an Association for Assessment and Accreditation of Laboratory Animal Care (AAALAC) approved facility, adhering to Swiss laws and regulations for animal protection. A total of 16 skeletally mature (2 to 4 years old) female Swiss Alpine sheep were included in this study. Prior to enrolment, each sheep underwent a physical examination and radiographic screening to ensure the tibial canal accommodated the intramedullary nail. In addition, a complete blood count was obtained from the internal jugular vein to exclude the presence of preoperative inflammation. The sheep were group housed for at least 2 weeks before surgery to acclimatize to the housing conditions. They were kept in an environment with daily cycles of 12 hours of light and dark and were fed twice per day with hay fed ad libitum, a mineral lick, and hand-fed grain.

**Figure 1:**
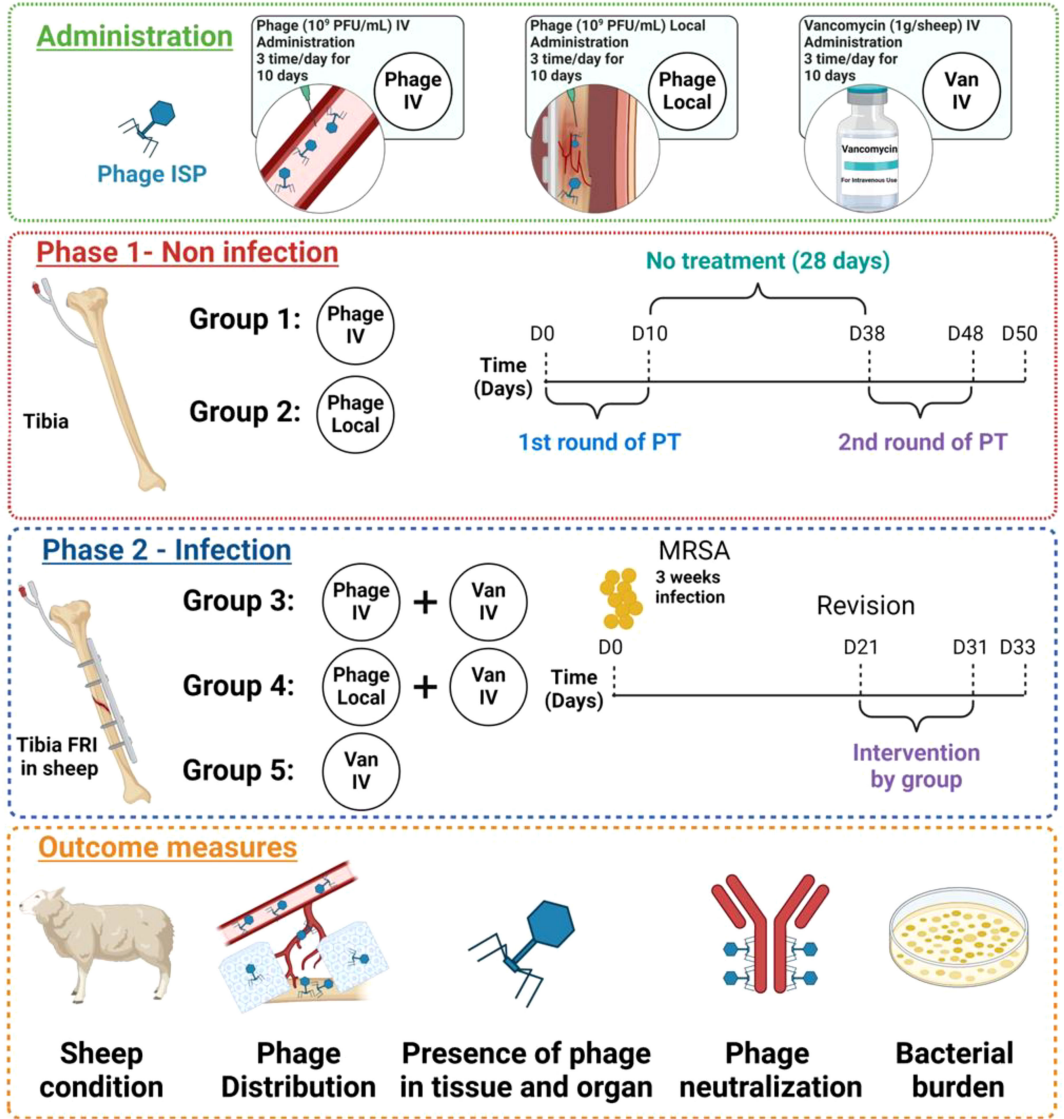
Overview of study design. Phased experimental protocol for administering phage therapy (PT) and vancomycin (Van) in sheep, depicting both non-infection and infection phases. The green box describes three treatments, including phage intravenous administration, phage local administration, and vancomycin intravenous administration. The red box is Phase 1 (Non-infection): Groups receive treatments by specific administration routes, Group 1 received intravenous phage administration, and Group 2 received phage instillation through a drainage tube (local). Groups 1 and 2 initially received 10 days of PT (1st round), followed by a subsequent 28-day no-treatment interval. This is then succeeded by a second 10 days of PT (2nd round), concluding on Day 50. The blue box is Phase 2 (Infection): The sheep included in this phase received osteotomy, plate and screw osteosynthesis, and were infected with MRSA MSI-003 at day 0. Revision surgery was performed at day 21 after which they received a combination of intravenous phage and intravenous vancomycin for group 3, a combination of local phage and intravenous vancomycin for group 4, and intravenous vancomycin only for group 5. These treatments continued for 10 days, followed by a two-day wash-out period, leading up to a final evaluation through euthanasia and sample collection at day 33. The orange box is outcome measures, including the general condition of the sheep, the distribution of phage within the body, the presence of phage in tissues and organs, the bacterial burden in infected areas, and the level of phage neutralization.

The study was conducted in two phases: Phase 1 assessed phage tolerability, pharmacokinetics, and neutralization in non-infected sheep, while Phase 2 evaluated all of the above plus the safety and efficacy of PT in treating MRSA-induced FRI. An overview of the study design is shown in **Figure 1**.

Sheep in Phase 1 did not have any fracture or infection but had a filtration probe to collect fluids in the intramedullary canal of the tibia. Twenty mL of phage ISP (10^9^ PFU/mL) suspension was administered in two rounds, three times per day, each lasting 10 days, with a 28-day interval between each round. The phage titer was adapted from previous studies of PT in *S. aureus* infections in rabbit and mouse models (Wills et al., 2005; Capparelli et al., 2007; Kishor et al., 2016). Group 1 (n=3) received phages intravenously (IV), and Group 2 (n=3) received phages by instillation through drainage (local). All sheep received an identical total phage titer regardless of route of administration. The animals were housed for an additional 2 days (a total of 50 days) after the cessation of the second PT to continue drawing serum samples and measuring residual phage and phage neutralization after euthanasia.

Sheep in Phase 2 had an FRI of the tibia (in the setting of plate fixation) caused by a human-isolated *S. aureus* (MRSA MSI-003). After three weeks, a revision surgery was conducted, which involved debridement and irrigation, as well as the placement of filtration probes within the intramedullary canal of the tibia. Following the revision surgery (on day 21), all sheep in phase 2 received systemic treatment with vancomycin administered as an intravenous infusion over the course of one hour, three times daily, for a duration of ten days (1g per sheep, Sandoz Pharmaceuticals AG, Rotkreuz, Switzerland). Sheep in phase 2 were divided into 3 groups, (n=3 per group): Group 3 received 20 mL of phage ISP (10^9^ PFU/mL) intravenously; Group 4 received 20 mL of phage ISP (10^9^ PFU/mL) locally; group 5 served as the control group without any phage application. Both groups 3 and 4 received phages three times daily for 10 days. After a two-day washout period to prevent false negatives, the sheep were euthanized 33 days after the first surgery which involved the induction of the FRI.

### 2.3 Surgical procedure and group allocation

#### 2.3.1 Phase 1

After anaesthesia and aseptic preparation of the right tibia, the intramedullary (IM) canal was exposed through an intramedullary nailing approach medial to the patellar ligament. A guide wire was inserted at the anterior edge of the tibial plateau, aiming down the tibial crest and thus the centre of the IM canal. The tibial cortex was opened using a cannulated awl over the previously placed guide wire, followed by insertion of a custom-sized filtration probe (Bioanalytical Systems, West Lafayette, IN) into the in IM canal under fluoroscopic guidance (**Supplementary Figure S1**). For those sheep receiving local PT, a separate drainage system (Bioanalytical Systems, West Lafayette, IN) was installed through the same incision and tunneled to exit laterally. The probes were secured with Chinese finger trap sutures, and the area was closed in three layers with absorbable suture material (#3-0 non-cutting and cutting Monocryl, Ethicon), followed by protective gauze and bandages. Group 1 (n=3) received phage ISP (20 mL, 10^9^ PFU/mL) intravenously, and Group 2 (n=3) received phage ISP (20 mL, 10^9^ PFU/mL) locally, both treatments being administered three times daily for 10 days. The first round of PT involved replacing the collection drainage bottle of the ultrafiltration probe three times daily for 10 days, followed by storage at 4°C. Post-therapy, all devices were removed, followed by bacterial quantification to exclude bacterial contamination. After four weeks, revision surgery was performed, involving similar preparatory steps to re-expose the tibial area and adjust or reinstall the necessary medical devices. This phase included two 10-day rounds of PT, separated by a 28-day interval without treatment, spanning from Day 0 to Day 50.

#### 2.3.2 Phase 2

The preparatory steps for Phase 2 were similar to those in Phase 1, with additional surgical procedures to secure a 10-hole, 5.5 mm steel plate (DePuy Synthes) on the tibia with locking screws and perform a transverse osteotomy with a 2 mm gap, which was inoculated with 1mL of MRSA MSI-003 (2x10^6^ CFU/mL) to induce infection (Siverino et al., 2023). Post-surgery, sheep were supported in slings to reduce stress on the tibia. After 21 days, the surgical site was reopened, a formal debridement was performed to remove infected tissue for analysis, and the plate was retained. The bone and implant were flushed with 1 L of saline (0.9%) and fluid was collected for bacteriological culture. The filtration probe and drainage system setup (**Supplementary Figure S1**), as described in Phase 1, was applied to Group 3 (n=3) and Group 4 (n=3). Group 3 received a combination of intravenous phage ISP (20 mL, 10^9^ PFU/mL, 3 times) and intravenous vancomycin (1g per sheep every 8 hours). Group 4 received local phage ISP (20 mL, 10^9^ PFU/mL, 3 times) with intravenous vancomycin (1g per sheep every 8 hours), while Group 5 (control, n=3) received only intravenous vancomycin (1g per sheep every 8 hours). PT was scheduled 21 days after initial surgery, and assessments were conducted until Day 33.

### 2.4 Phage preparation

ISP, a single phage targeting *S. aureus* was used with permission from the Eliava Institute, Georgia. The ability of ISP to infect MRSA MSI-003 was confirmed. Phage preparation was conducted using the double-agar overlay method. Initially, the bacterial strain was cultured overnight in TSB broth at 37°C. Bacteriophages were then diluted in Dulbecco phosphate buffer saline (DPBS, [2.7 mM Potassium Chloride (KCl), 1.5 mM Potassium Phosphate monobasic (KH2PO4), 137.9 mM Sodium Chloride (NaCl), 8.1 mM Sodium Phosphate dibasic (Na2HPO4-12H2O)]) buffer and 100 µL of the phage dilution was mixed with 100 µL of bacterial suspension (MRSA MSI-003), combined with 4 mL of TSA soft agar (3% TSB and 0.6% agar), and poured onto TSA plates. These plates were incubated at 37°C overnight. After incubation, the phages were harvested by covering the top layer of the plates with 5 mL of DPBS and placing them overnight at 4°C. The upper liquid layer was then transferred into a 50 mL Falcon tube. The suspension was centrifuged at 3260 rpm, 4°C for 10 minutes, and the supernatant was subsequently filtered through 0.45 µm and then 0.22 µm filters (Millex, Merck Millipore, Ireland). To further purify and concentrate the phages, the supernatant was filled in Spectra/Por^®^ 7 dialysis membranes with 100,000 Da molecular weight cutoff (Spectrum Laboratories, Inc., Rancho Dominguez, CA, USA) to dialyze over three days at 4°C. Ultrafiltration was then performed using an Amicon filter (Millipore Sigma) with a 100 kDa cut-off. The phage titer was finally determined using a double agar overlay method to ensure the titer was appropriate (10^10^ PFU/mL) for subsequent experimental applications.

### 2.5 Phage pharmacokinetics and quantification *in vivo*

For Phase 1, blood samples (10ml) from Groups 1 and 2 were collected at 5, 30, 60, 120, 240, and 360 minutes after phage administration on days 1, 5, and 10 during first round of PT, and on days 38, 42, and 47 during second round of PT via a central venous catheter in the internal jugular vein. For Phase 2, blood samples from Groups 3, 4 and 5 were collected at 5, 30, 60, 120, 240, and 360 minutes after phage administration on days 1, 5, and 10 during PT. The active phage titer was determined by the double agar overlay method. Briefly, an aliquot of blood was centrifuged at 3220 × g for 10 min at 4°C to pellet cells and debris. The plasma was collected and filtered through a 0.45 µm filter and then through a 0.22 µm to remove debris and bacteria. Subsequently, 100 µL of the filtrate was mixed with 100 µL of bacterial suspension (MRSA MSI-003) and added to 4 ml of soft agar. The mixture was transferred to the TSA plate after gently mixing and we counted the plaque-forming units after overnight incubation in a 37 °C incubator. All experiments were performed in triplicate.

Routine evaluation of phage titer entailed the daily collection of plasma and draining fluid samples each morning prior to the commencement of daily PT to monitor baseline phage titers. At euthanasia, additional titers were taken from tissues as outlined below. Sheep were euthanized at day 50 for Phase 1 and at day 33 for Phase 2. The bone (i.e. osteotomy, proximal bone, distal bone and bone marrow), soft tissue and organs, including lung, kidney, liver, spleen, iliac lymph nodes, and popliteal lymph nodes were harvested (approximately 20g total for each sample). Three samples were randomly taken from each organ and homogenized (Omni TH, tissue homogenizer TH-02/TH21649) in 20 mL of sterile PBS. The plate and screws were removed from the bone, transferred into a glass tube containing 20 ml PBS and sonicated in an ultrasonic water bath (Model RK 510H, Bandelin electronic GmbH & Co. KG, Berlin, Germany) for 10 min. An aliquot of each tissue sample and sonication fluid from the implant was centrifuged at 3220 × g for 10 min at 4°C to pellet cells and debris. The plasma, draining fluid and supernatant were collected and filtered through a 0.45 µm filter and then through a 0.22 µm filter (Millex, Merck Millipore, Ireland) to remove debris, bacteria and small contaminants. The active phage titer was determined by the double agar overlay method as mentioned above.

### 2.6 Phage neutralization assay

Phage neutralization in the serum was determined by a modified version of the phage-neutralization assay as previously described (Chen et al., 2023). Briefly, 900 µL of serum, diluted at a 1:100 ratio, was mixed with 100 µL of phage ISP (10^7^ PFU/ml). This mixture was incubated at 37 °C for 30 minutes. Following incubation, the mixture was further diluted by a factor of 1000 using 0.9% saline. The phage titer was then determined using the double-agar overlay technique as outlined above. The percentage of phage particles neutralized was calculated using the formula: (P0 - P1)/P0 × 100%, where ‘P1’ represents the phage titer post-serum incubation at the time of euthanasia, and ‘P0’ was the titer after incubation with serum obtained before the initial surgery.

### 2.7 Bacterial quantification

The bone (i.e. osteotomy, proximal bone, distal bone and bone marrow), soft tissue adjacent to the plate and bone and organs, including lung, kidney, liver, spleen, iliac lymph nodes, and popliteal lymph nodes were collected in Phase 2 at euthanasia to quantify bacterial load. In particular, the bacterial load was examined for the FRI site separately for its subdivisions - soft tissue, osteotomy, and implant - to assess the overall effect of the phage therapy and its efficacy in different tissues/materials. These samples underwent ten-fold serial dilutions and plated on 5% horse blood agar plates (BA, Oxoid Ltd., Hampshire, United Kingdom). The plates were incubated at 37°C, and bacterial colonies were quantified after 24 hours. Plates were also monitored at room temperature for another 24 hours to check for slow-growing colonies or potential contaminants by Staphaurex plus latex agglutination test (Thermo Scientific).

### 2.8 Statistical analysis

All experiments were conducted in triplicate, with each test being repeated three times, and the results were expressed as mean ± standard deviation. Descriptive and statistical data analysis was performed and visualized using GraphPad Prism 9 (GraphPad Software). The Shapiro-Wilk test was employed to assess the normality of continuous data, while the homogeneity of variances was evaluated using Levene’s test. For parametric data, group differences were analyzed using Student’s t-test or a one-way ANOVA. For nonparametric data, the Kruskal-Wallis test or Mann-Whitney U test was applied. Tukey posttest was performed to compare more than two groups. For all tests, p-value < 0.05 was considered statistically significant.

## 3 Results

### 3.1 Phase 1: phage therapy for non-infected sheep

#### 3.1.1 Animal welfare and safety

Six sheep were included in Phase 1. Postoperatively, the sheep recovered well after each surgery. In general, there was no significant difference in weight change between the two groups. Both groups maintained stable weights throughout the study, with no notable weight loss observed (**Supplementary Figure S2A**). Moreover, no distinct pattern could be observed for white blood cell counts (WBC) between two groups at each time point (**Supplementary Figure S2B**).

#### 3.1.2 Phage titer in plasma in healthy sheep

**Figure 2:**
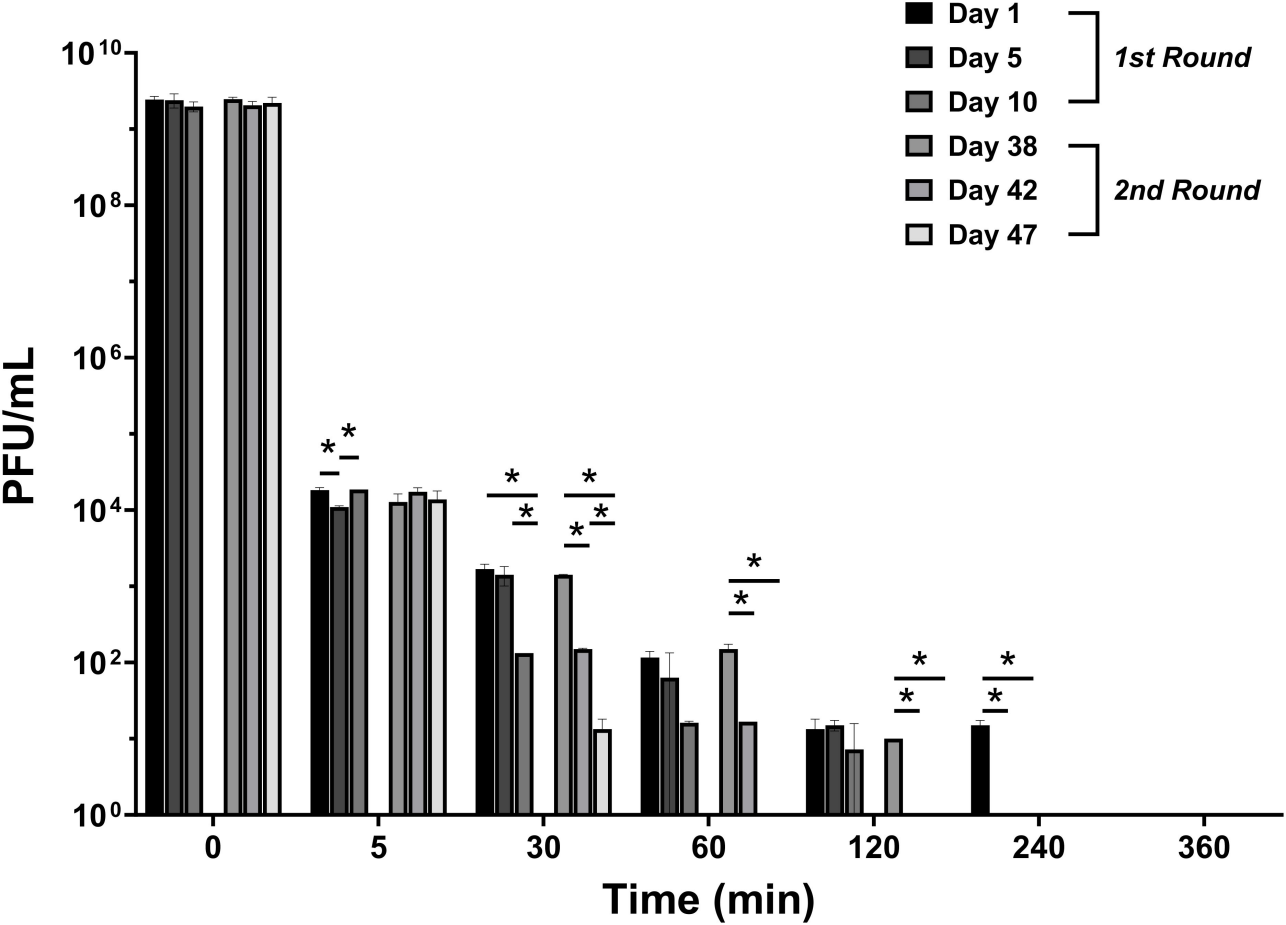
Phage distribution in serum after the first and the second round of intravenous administration. Measurements were taken at 5, 30, 60, 120, 240, and 360 minutes. The titer of phage administered intravenously and locally was 10^9^ PFU/mL for both. PFU, Plaque-forming unit. The data were presented as mean ± standard deviation of results, and statistical significance was determined using a one-way ANOVA test followed by Tukey posttest, *p < 0.05. The data for the local administration are not shown in this figure as phage titers were below the detectable limit. The detection limit of the assay used to quantify active phage was 10 PFU/mL.

In case of intravenous administration, a rapid decline (4 log_10_ reduction) was observed within the first 5 minutes and this rapid decrease continued, leading to undetectable levels within 6 hours (**Figure 2**). A similar trend of phage distribution was observed in the second round of PT given intravenously, although phages reached undetectable levels sooner, within 4 hours (**Figure 2**). The decrease in active phage particles was even more pronounced after several administrations had been performed in the second round of applications (**Figure 2**).

The distribution of the phage in the serum was also analyzed following local administration, however, no active phage was detected in the serum at any time over a period of 6 hours.

#### 3.1.3 Phage titers in tissue in healthy sheep

No viable phages were detected in the draining fluid or plasma collected before daily PT administration for either intravenous or local administration during the first and second rounds of therapy, as determined by the double agar method. Additionally, during the final evaluation after euthanasia, no viable phages were found in the soft tissue, bone, implant, lung, kidney, liver, spleen, iliac lymph nodes, or popliteal lymph nodes for either intravenous or local administration, as confirmed by the double agar method.

#### 3.1.4 Phage neutralization in healthy sheep

Initially, the serum from healthy sheep with both intravenous and local administrations showed a rapid increase in presumptive anti-phage antibodies (indicated by neutralized phage particles) after the first round of PT, with levels peaking at approximately 60% by day 10. During the subsequent 28-day no-treatment period, local administration showed a significant decline (*p*<0.05) in phage neutralization, dropping to approximately 30% by day 24 and further decreasing to approximately 20% by day 38. In contrast, the intravenous administration maintained a relatively stable phage neutralization during the no-treatment period, stabilizing at approximately 50% by day 38.

**Figure 3:**
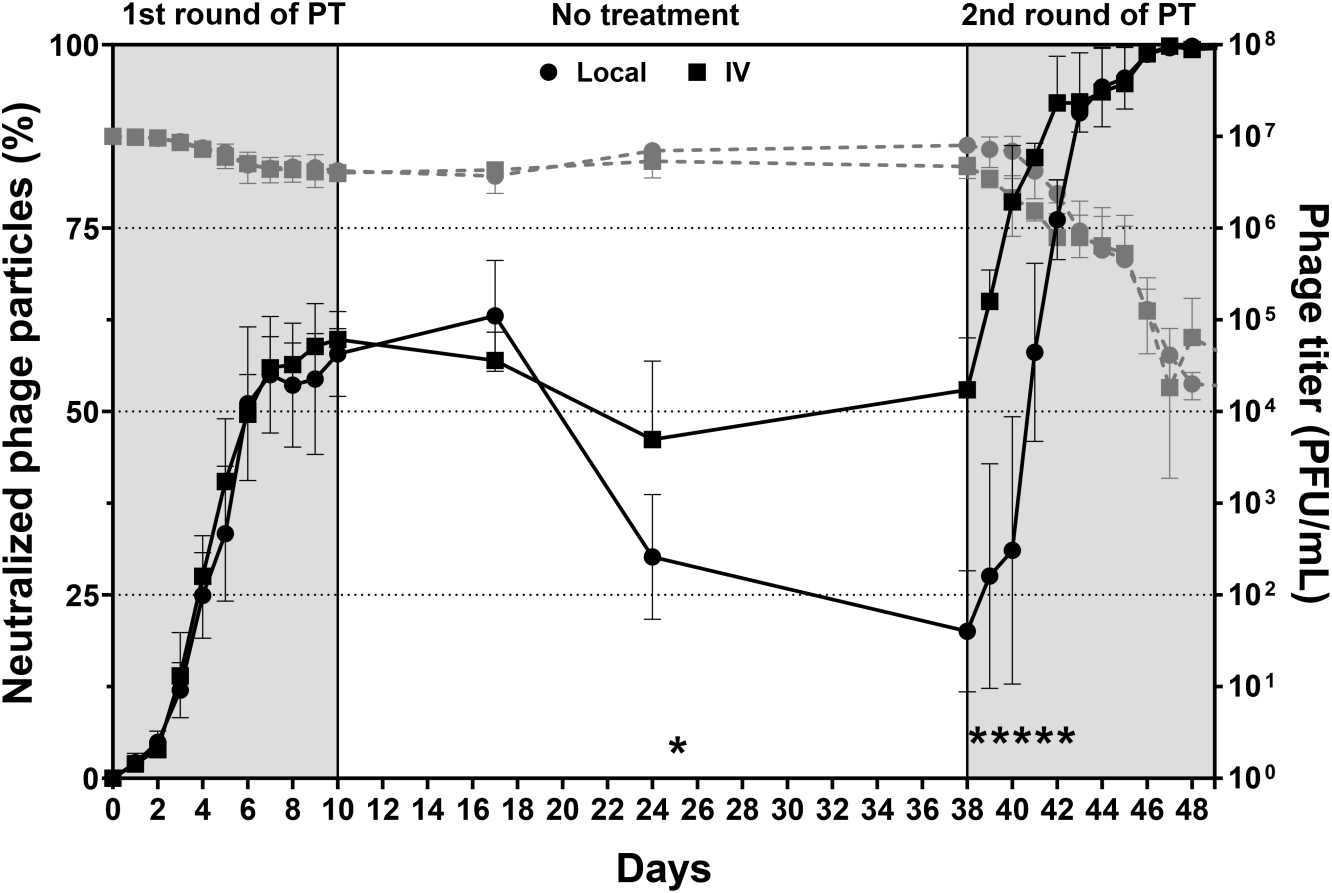
Phage neutralization between intravenous and local administrations. The black solid line represented the percentage of phage neutralization and should be read on the left Y-axis. The grey dashed line represented the phage titer of phage neutralization and should be read on the right Y-axis. PT was administered over two 10-day treatment periods separated by a 28-day no-treatment period. Higher neutralization values indicated reduced phage activity. The data are presented as mean ± standard deviation, and statistical significance was determined using a student’s t-test (*p < 0.05). The detection limit of the assay used to quantify active phage was 10 PFU/mL.

The second round of PT, beginning at day 38, showed a rapid increase in phage neutralization for both administration routes, from days 38 to 42, with IV administration demonstrating a significantly faster increase in phage neutralization compared to local administration. It rose to approximately 99.9% by the end of the second round on day 48 and was broadly equivalent for both local and IV at this time (**Figure 3**).

### 3.2 Phase 2: phage therapy for infected Sheep

#### 3.2.1 Animal welfare and safety

Nine sheep were planned for inclusion in Phase 2. One sheep from the vancomycin-only control group died due to oesophageal obstruction, and a replacement sheep was introduced. All sheep recovered well postoperatively after each surgery. In general, there was no consistent difference regarding weight change or WBC counts among the 3 groups throughout the study duration (**Supplementary Figure S3**). However, a slight increase in WBC counts was observed at the revision time point (d21) in animals that received phage IV and vancomycin IV, as shown in **Supplementary Figure S3B**. This increase was not statistically significant, though it may indicate a response to the surgical procedure or the infection status at that point (**Supplementary Figure S3B**).

#### 3.2.2 Infection burden after phage therapy

**Figure 4:**
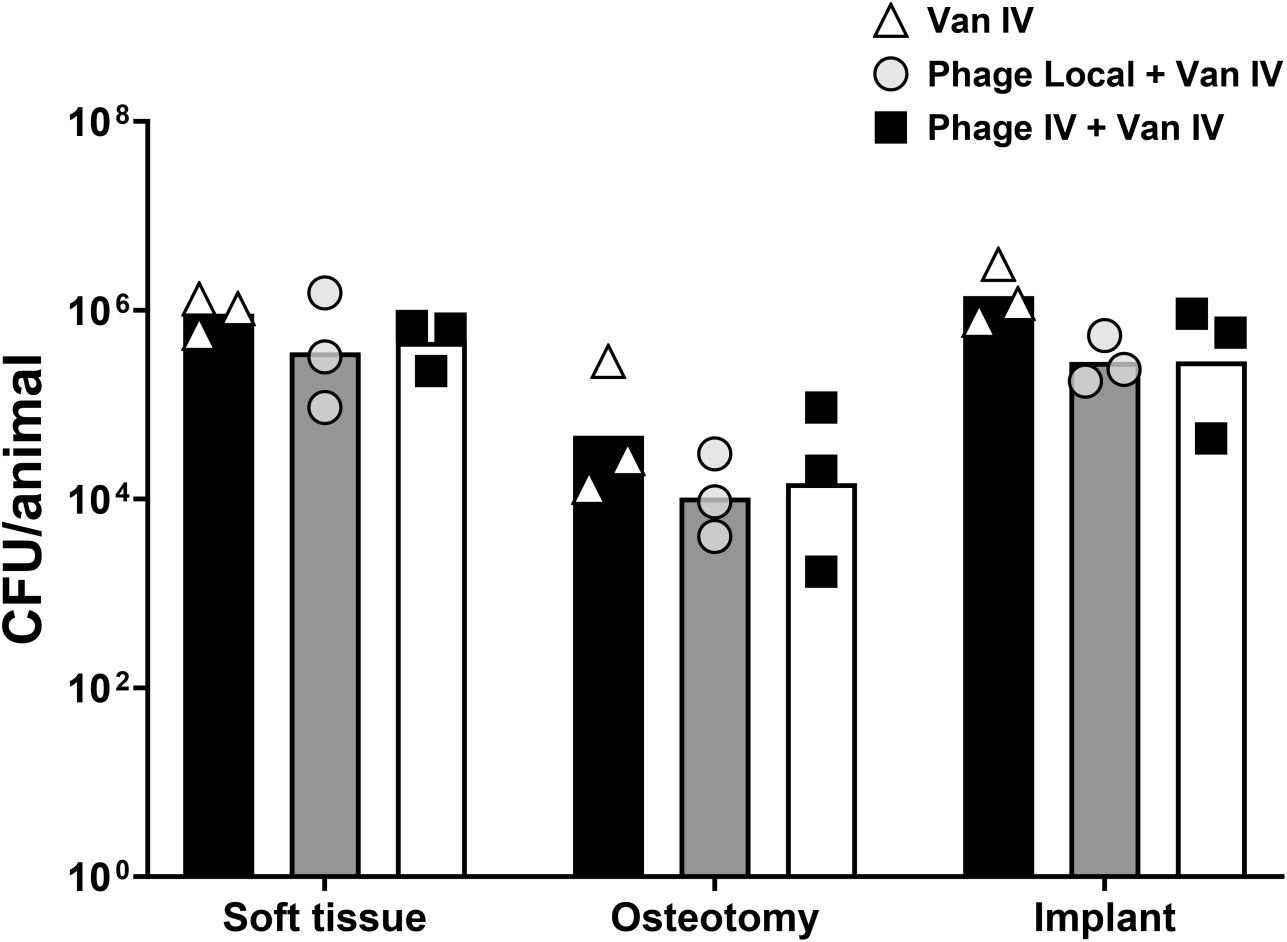
Bacterial load in soft tissue, osteotomy, implant, and total bacterial load. Sheep were treated with either vancomycin alone, intravenous phage therapy combined with intravenous vancomycin, or local phage therapy combined with intravenous vancomycin. Data represent the mean ± standard deviation, and statistical significance was determined using a Kruskal-Wallis test followed by Tukey post test. CFU, colony forming units.

The bacterial load at euthanasia was assessed across different tissue locations: soft tissue, osteotomy, and implant, for each animal. In the soft tissue, osteotomy site and implant, both PT routes achieved variable bacterial load reduction when compared to sheep receiving vancomycin only, however, the differences were not statistically significant (**Figure 4**). Additionally, bacterial load was evaluated across distinct anatomical segments of bone, including the proximal and distal regions as well as the bone marrow. However, no significant difference was observed across different bone locations (**Supplementary Figure S4**).

#### 3.2.3 Phage titer in plasma in infected sheep

**Figure 5:**
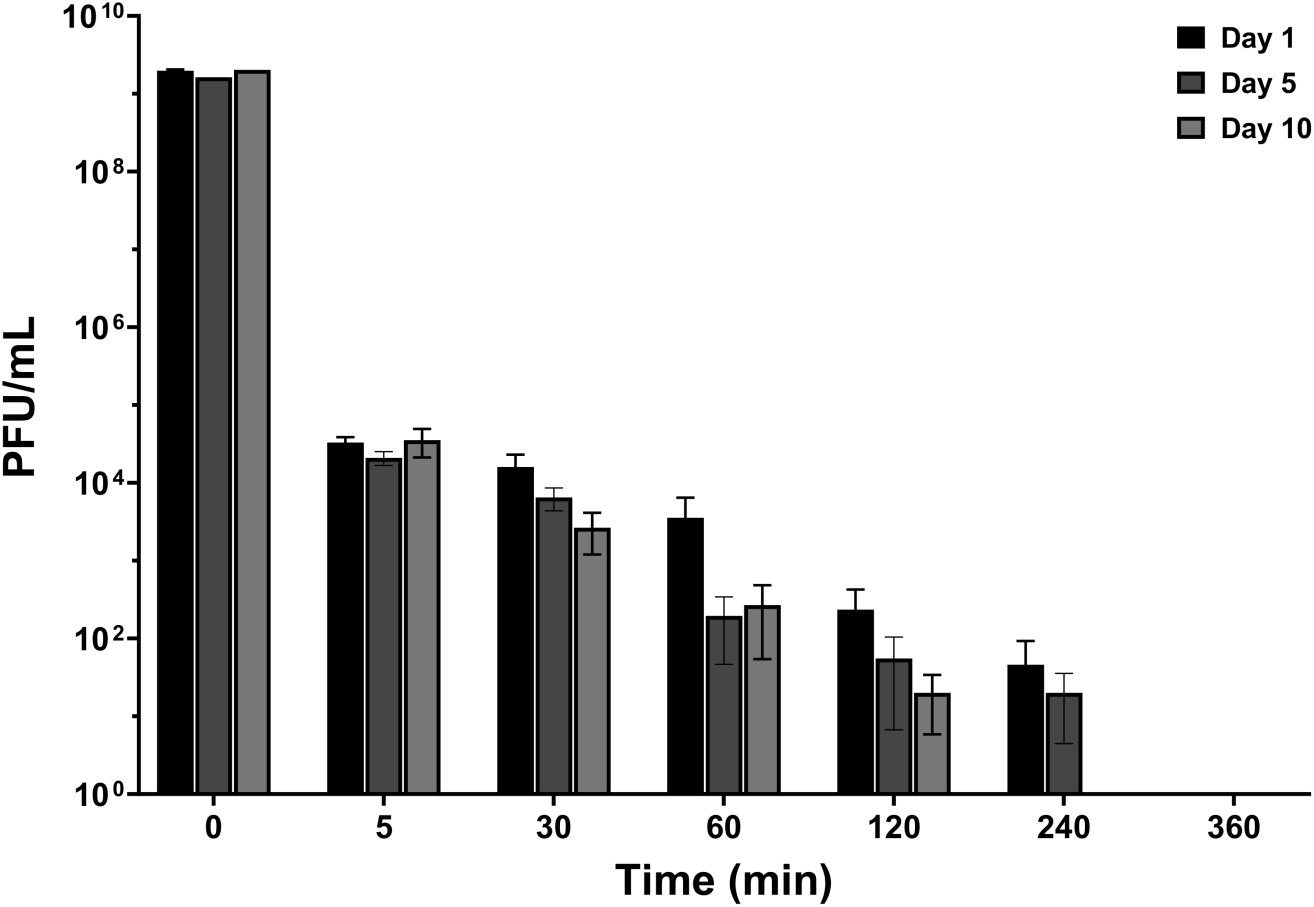
Phage distribution in plasma after intravenous administration and local administration. Measurements were taken at 5, 30, 60, 120, 240, and 360 minutes on days 1, 5, and 10 post-PT. The titer of phage administered intravenously and locally was10^9^ PFU/mL. The data were presented as mean ± standard deviation of results and error bars represent standard deviation, and statistical significance was determined using a one-way ANOVA test followed by Tukey posttest. The detection limit of the assay used to quantify active phage was 10 PFU/mL.

The phage titer in the plasma of sheep subjected to local PT combined with intravenous vancomycin, as well as sheep administered intravenous PT combined with intravenous vancomycin, was assessed to determine the distribution of phages. There was a significant reduction in phage concentration (4 log_10_) within the first 5 minutes after intravenous PT, and a continued rapid decline in phage concentration within the first few hours post-therapy, leading to near-undetectable levels within 6 hours (**Figure 5**). There was no significant change in active phage count after repeated phage administration (day 5 and 10) compared with the first administration (day 1). Moreover, no active phage was detected in the plasma at any time during the 6 hours after local PT.

#### 3.2.4 Phage titers in tissue in infected sheep

No viable phages were detected in the draining fluid or plasma collected before daily PT administration for either intravenous or local administration during PT, as determined by the double agar method. Additionally, during the final evaluation after euthanasia, no viable phages were found in the soft tissue, bone, implant, lung, kidney, liver, spleen, iliac lymph nodes, or popliteal lymph nodes for either intravenous or local administration, as confirmed by the double agar overlay method.

#### 3.2.5 Phage neutralization in infected sheep

**Figure 6:**
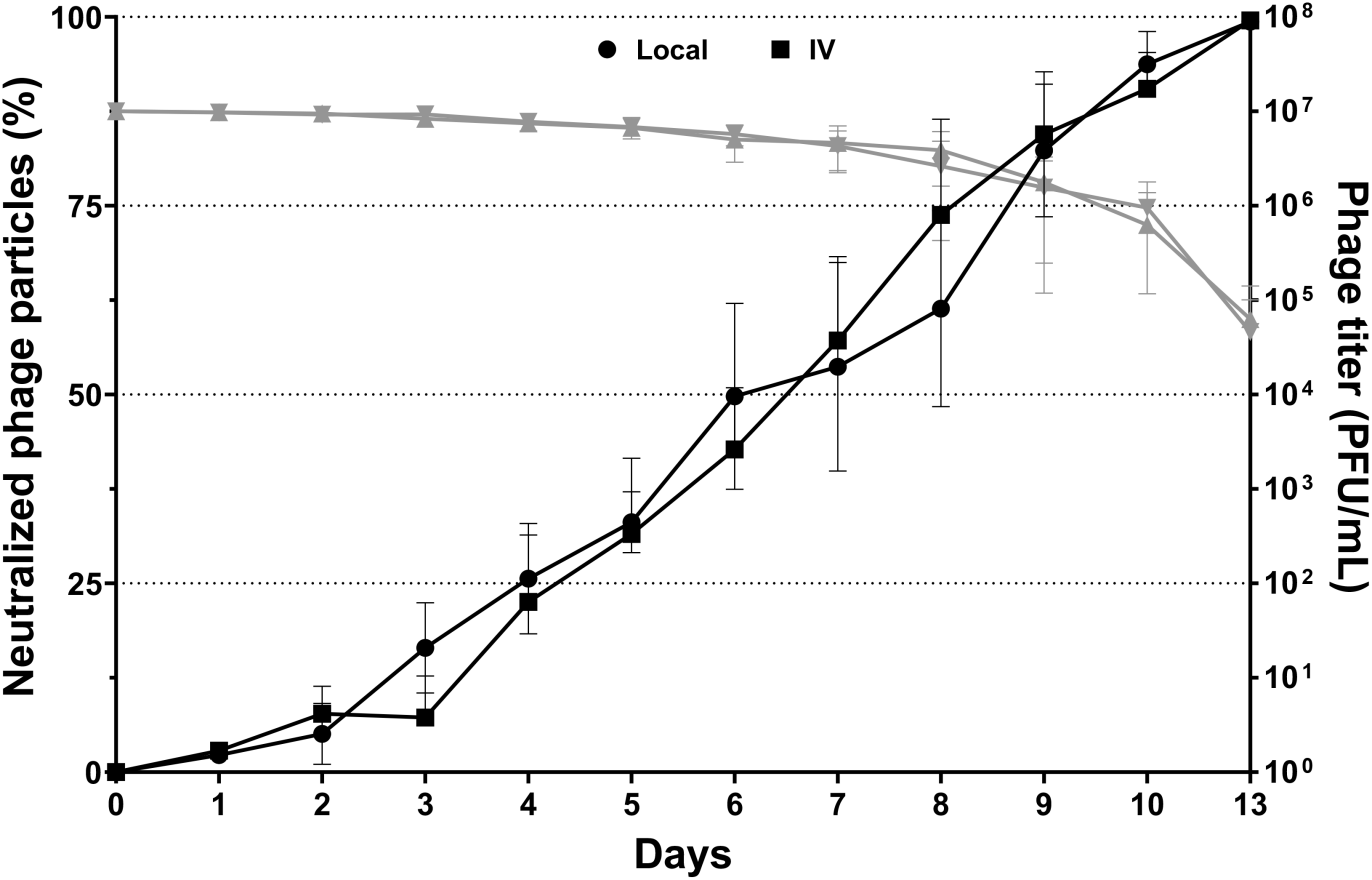
Phage neutralization between intravenous and local administrations over a 13-day period. Error bars indicate the standard deviation of the measurements. The black solid line represented the percentage of phage neutralization and should be read on the left Y-axis. The grey dashed line represented the phage titer of phage neutralization and should be read on the right Y-axis. The data were presented as mean ± standard deviation of results and error bars represent standard deviation, and statistical significance was determined using a student’s t-test (*p < 0.05). The detection limit of the assay used to quantify active phage was 10 PFU/mL.

Initially, both administration routes resulted in a gradual increase in phage neutralization. By day 3, local administration reached approximately 16.4% neutralization, while intravenous administration reached approximately 7.2%. As the experiment progressed, both administration routes resulted in an increase in phage neutralization. By day 7, local administration reached approximately 53.7% neutralization, and intravenous administration approximately 57.2%. The trends continued upward, with local administration reaching about 93.7% and IV administrations at about 90.5% by day 10 at the end of PT. After a 3-day hiatus without any treatment, both administrations reached their peak in phage neutralization by day 13: local administration reached approximately 99.4% neutralization, while intravenous administration reached approximately 99.5%. No statistical differences were observed between the two administrations at each time point throughout the experiment (**Figure 6**).

## 4 Discussion

This study mimicked a challenging human case of FRI treated with PT in a controlled *in vivo* preclinical study, using the same pathogen and phage used in PT. In human cases, significant clinical improvement has been observed, underscoring the potential of PT as a valuable adjunct to conventional antibiotic treatment. However, although such positive clinical outcomes exist for PT in human patients, there are still unanswered questions regarding the optimal application, safety, pharmacokinetics, and efficacy of PT. The large animal study was, therefore, designed to answer some of these fundamental open questions.

Overall, our findings revealed some important information about PT and how it performs in a controlled preclinical model. PT was found to be safe, as evidenced by the stable weight of sheep across the study, the absence of significant differences in WBC counts among the treatment groups, the lack of observable side effects, and the absence of active phages in organs. The rapid elimination of phage from the circulating plasma and an inability to detect active phage locally during treatment were primary outcomes. The lower-than-expected titers of phages may be a result of a combination of factors including rapid induction of neutralizing antibodies, and dilution in a large animal as will be discussed below. Secondly, we did not see any significant additional antibacterial benefit in adding phages to systemic antibiotic therapy. Although trends were observed for a reduction in CFU, the effect was insufficient to eradicate the infection.

Intravenous administration is efficient and very fast in terms of phage delivery. However, our results demonstrated a rapid decline in phage titer in the plasma following intravenous administration. This rapid clearance suggests that phages may be diluted in plasma or quickly removed from circulation. It was observed in a rabbit study that immediately after injection (within 2.5 minutes), the phage concentration in rabbit blood was 100 times lower than the expected titer, which was calculated based on the dose and expected dilution in the blood volume (Sulkin et al., 1957). In the context of our study, using sheep which most closely approximate the human scale, the expected phage titer following intravenous injection was calculated to provide a theoretical baseline for comparison. For example, in a sheep weighing 70 kg, the blood volume was estimated based on a standard value of 60 mL/kg of body weight (Gillett and Halmagyi, 1966), resulting in a total blood volume of approximately 4200 mL. Given an initial phage dose of 20 mL ×10^9^ PFU, then the expected phage titer was determined to be approximately 4×10^6^ PFU/mL. However, the phage titer in the blood immediately after injection (5 minutes) is approximately 10^4^ PFU/mL. This is markedly less than expected from the hypothetical dilution of phage in body volume (100-fold lower) and it suggests a very rapid mechanism of capturing or neutralizing the phages in sheep.

Liver and spleen are the most commonly reported organs that accumulate phage delivered systemically (Dabrowska and Abedon, 2019). As important elements of the mononuclear phagocytic system (MPS), they filter many foreign objects traveling in circulation, including phages (Van Belleghem et al., 2018). Typically, the spleen is the organ where active phages can be detected for the longest time, even for 12 days after administration in rabbit (Reynaud et al., 1992). However, in our study, no active phage was observed in the sections of organs that were tested at euthanasia. It is possible that phages were present in untested areas or remained below the detection limit of our assay. It could indicate that the phages were rapidly cleared or inactivated by the host immune system before they could accumulate in these organs. The rapid phage clearance, seen in plasma, suggests that the immune system played a major role in this process. Furthermore, the absence of detectable phages could be attributed to the utilization of the double agar overlay method, which exclusively identifies active phages. In contrast, polymerase chain reaction (PCR) techniques are capable of detecting both active and inactive phages. Given that PCR was not employed in this study, it is plausible that inactive phages were present in the organs. Moreover, considering that three days had passed without any PT prior to euthanasia, it is plausible that the phages had been eradicated within the body during this washout period. This is more relevant for the non-inoculated sheep, where the bacterial host is absent so replication of phage was not possible, and only the introduced phage could be expected to be present.

Not only was the phage titer in plasma lower than may have been expected, phages were the not even detected in draining fluid and bone 72 hours after last administration upon euthanasia. This may be attributed to suboptimal distribution resulting from delivery via the drainage. In the sheep model, phages were administered locally through this tube to concentrate them at the infection site. However, this method might not have allowed adequate dispersal throughout the target tissue. This is likely a limitation of the experimental setup, rather than a broader issue with phage therapy itself. Improvements in phage delivery systems, such as for example via the use of continuous infusion or application in biomaterials, could enhance tissue penetration in future studies.

One of the key host responses influencing the outcome of PT is the induction of a specific immune response to the phage itself. In healthy sheep, phage neutralization reached approximately 60% by day 10 and dropped significantly after the first PT round but surged to 99.9% upon re-exposure in the second round. This suggests that the immune system retains memory of the phages and can respond robustly upon subsequent exposure, even without infection. In the presence of bacteria as modelled in phase 2 with MRSA infection, neutralization rose even more quickly, reaching 99% by day 13 at the end of PT. This indicates that ongoing bacterial presence boosts immune activity against phages and was not different for local or IV administered phages.

Rapid induction of phage neutralization may cause a rapid decline in phage titer, which leads to an insufficient bacterial load reduction. Phage neutralization levels in our sheep FRI models peaked at 99% by day 10. In contrast, previous studies in human patients showed phage neutralization occurring between 6 and 35 days after starting therapy in 38.5% of cases, particularly with invasive phage administrations (Pirnay et al., 2024). Two case reports found no phage neutralization in patients with FRI during local phage treatment (Onsea et al., 2021a; Eskenazi et al., 2022). Similarly, Cano et al. observed IgG anti-phage antibodies in periprosthetic joint infection patients but saw no significant changes in IgG levels over time (Cano et al., 2021). The difference in phage neutralization between humans and sheep, despite identical administration and therapy duration, is possible due to variations in the immune response. Many patients suffered from long-term osteomyelitis before receiving PT, possibly altering the immune response (Surendar et al., 2024). In chronic infections, immune tolerance and exhaustion involve regulatory mechanisms that suppress the immune response to minimize inflammation and tissue damage (Rogovskii, 2020). This creates a less aggressive immune environment, potentially allowing therapeutic agents like phages to act with less interference from the host’s defenses. In contrast, sheep models, with their shorter infection duration, perhaps had a more active and less tolerant immune system, that could result in higher phage neutralization due to a vigorous immune response to both bacterial pathogens and therapeutic phages (Nishitani et al., 2016). This could also explains why the efficacy outcome is not positive. Conversely, in humans with chronic infections and associated suppressed or reduced immune responses, phage neutralization might be less of an issue, as evidenced by improved bacterial clearance and therapeutic outcomes (Onsea et al., 2021b; Uyttebroek et al., 2022; Metsemakers et al., 2023b). For instance, in one case study, a 15-year-old lung transplant patient, likely due to rituximab treatment, did not develop phage neutralization (Dedrick et al., 2019). Another patient on photopheresis, intravenous immunoglobulin, sirolimus, and prednisone also exhibited immune tolerance, likely lowering phage neutralization risk (Dan et al., 2023). This suggests that phage neutralization is a key consideration in PT, but its impact can vary significantly based on the immune status of the patient. To enhance PT outcomes for FRI, a tailored approach considering immune status is beneficial. Pre-therapy immune assessments could evaluate markers of tolerance and exhaustion to predict phage neutralization risks. Optimizing phage formulations with immune modulators, protective carriers, or selecting phages with inherently low immunogenicity can help reduce immune exposure and extend therapeutic effects based on these findings. By integrating these strategies, clinicians can enhance the efficacy of PT, particularly in patients whose immune systems may otherwise compromise the treatment’s success.

One of the challenges associated with phage neutralization in human PT is the potential for neutralization to persist long after the therapeutic intervention. Łusiak-Szelachowska et al. reported that patients with bone infections undergoing PT can develop high levels of phage-neutraliziation. In their study, PT lasted between 24 to 54 days, and the antibodies persisted for several months to over a year after therapy, showing considerable variation in how long they took to decline (Lusiak-Szelachowska et al., 2022). In another study, Eskenazi et al. reported a 6-day course of PT for FRI caused by pan-drug-resistant *Klebsiella pneumoniae*. In their case, no phage neutralization was observed during the first 8 days. However, neutralizing antibodies began to develop between days 8 and 18 after phage administration, and by 161 days post-therapy, a significant neutralization rate of 87.13% was detected (Eskenazi et al., 2022). The researchers considered that phage-neutralizing antibodies were unlikely to have impeded the efficacy of the PT due to the short course of treatment. In this study, we assessed phage neutralization before the start of PT, with baseline levels recorded as ‘Day 0.’ These baseline measurements were compared to control phage titers to determine changes in phage neutralization over time. However, phage neutralization is expected to persist for an extended period and is an important factor to consider in PT as also confirmed in our sheep study.

The choice between local and intravenous phage administration remains a key question in phage therapy. Our study found no significant difference in efficacy between the two routes of administration. Although no significant therapeutic efficacy was observed for either route of phage administration, we did observe phage neutralization reaching high levels (>90%) for both groups, indicating the rapid immune response to phage treatment. Local administration offers the advantage of targeting the infection site directly. However, IV administration provides a rapid systemic approach to potentially address infections at multiple sites. Despite similar levels of phage neutralization between the two methods, local administration may still provide an advantage in cases where localized infections, such as FRI, are the primary concern. Local delivery may reduce the likelihood of rapid clearance or dilution in the bloodstream, though more research is needed to confirm this. There is no reason from this study to suggest that local administration should be avoided.

In terms of efficacy, our study revealed that while PT demonstrated some antibacterial activity, it fell short of eradicating the infection, even when PT was combined with systemic antibiotics. Several factors may have contributed to this outcome. The rapid neutralization of phages by the host immune system likely played a significant role, as evidenced by the high phage-neutralizing antibody levels observed during treatment. The method of phage delivery, whether intravenous or local, may have influenced the distribution and availability of active phages at the infection site. The lack of detectable phages in both plasma and tissue further underscores the challenges of achieving sufficient therapeutic concentrations *in vivo*. This variability in immune response highlights the importance of considering different phage types in phage therapy. Phages, being highly specific to their bacterial targets, may interact with the immune system differently based on their structure, size, and other factors. Although few studies have compared immune responses to different phages or phage cocktails, it is not clear how other phages may be affected, but that it is an area requiring further research. Moreover, critical differences in treatment protocols must be considered. In this study, infection was treated with implant retention, which is known to be suboptimal compared to protocols involving implant removal. If the referenced patient underwent implant removal along with PT, the clinical context would differ significantly from the experimental conditions in our study. For these reasons, it is not appropriate to directly compare the results of our preclinical study to those of individual clinical cases. Instead, the study aims to address broader questions of PT efficacy, safety, pharmacokinetics, and immunogenicity within a controlled experimental framework.

Sheep were chosen as the large animal model due to their established compatibility with human clinical instrumentation and standardized infection induction and management protocols. While other large animals such as pigs offer greater phylogenetic similarity, their logistical demands and associated costs outweigh the potential gains in physiological relevance for this proof-of-concept study. Our selection of sheep prioritized translational feasibility, ethical efficiency, and alignment with existing experience in our facilities.

Despite the valuable insights provided by this study, several limitations need to be addressed. The relatively small sample size in both phases is a limitation. Nonetheless, intravenous vancomycin combined with local and intravenous PT resulted in a 66% and 52% reduction respectively, in the total bacterial load of a human isolated multi-resistant pathogen, which may still be considered clinically relevant, despite the lack of statistical significance. However, Larger studies, including those with increased group sizes, extended follow-up periods, better monitoring of human patients, and potentially additional animal models such as pigs or non-human primates, are necessary to confirm the trends observed and to establish more robust conclusions regarding the efficacy and safety of PT in FRI. Second, the study focused on monophage therapy (Alispahic et al., 2020) and a specific bacterial isolate (MRSA MSI-003). While this approach ensured consistency in the results, it may not fully represent the broader spectrum of phage-bacteria interactions seen in clinical settings. The findings might differ with other phage strains or bacterial species, suggesting the need for further studies involving multiple phages and pathogens to evaluate the general applicability of the results. Second, an acute FRI (Inoculum for 3 weeks) was simulated rather than a chronic infection. The increased immune response in the sheep models highlights the difficulty of using PT for acute infections, as the immune system may neutralize the phages prematurely. Conversely, in chronic infections with reduced or suppressed individual immune responses, phage neutralization may be less of an issue, improving bacterial clearance and therapeutic outcomes, as has been shown clinically. However, this would require dedicated and substantial studies to confirm these hypotheses (Onsea et al., 2021b; Uyttebroek et al., 2022; Metsemakers et al., 2023b). Finally, our study did not explore the impact of using phage cocktails or combinations of different phages, whereas phage cocktails are commonly employed in clinical PT to minimize the emergence of bacterial resistance and broaden efficacy. We did not assess whether bacteria isolated post-mortem developed phage resistance, which represents a limitation of this study and should be considered in future investigations.

This study contributes to the growing body of evidence on the safety and pharmacokinetics of PT in treating FRI caused by MRSA. The results demonstrate that both intravenous and local administration of phages are well-tolerated and result in similar levels of phage neutralization when treating implant-associated infections and resulting biofilms, although neither route provided a significant advantage over standard antibiotic therapy in reducing bacterial load. The findings highlight the complexity of PT, particularly the challenges posed by phage neutralization and rapid clearance from the bloodstream. While PT holds promise as a complementary approach to antibiotics, its clinical efficacy may require further optimization, including the selection of more effective phage strains, adjustment of dosing regimens, and improved delivery methods to overcome immune-mediated inactivation.

## Data Availability

The original contributions presented in the study are included in the article/**Supplementary Material**. Further inquiries can be directed to the corresponding author.

## Supplementary material

Supplementary Figure 1 The placement of the probe and the collection tube for the animals that received local administration. The left side is phase 1, animal without any osteotomy or fracture-related infection, and the right side is phase 2, where the animals received an osteotomy, plate ad screw fixation and fracture-related infection. The animals that received systemic administration (i.e. no PT) did not receive any tube for phage application.

Supplementary Figure 2 The measurements of **(A)** weight and **(B)** white blood cell (WBC) count in sheep receiving phage administration locally and intravenously. Weights were normalized to the preoperative weights at the primary surgery and set at 100%. 1st OP: The beginning of PT. 1st PT: D9 is the timepoint when PT of the first round has been administered for 10 days; 2nd OP: 28 days after the end of PT, the second operation started at Day 37; 2nd PT: D47 was the timepoint when PT of the second round has been administered for 10 days. Data points represented individual measurements, with horizontal lines indicating the mean values and statistical significance was determined using a student’s t-test.

Supplementary Figure 3 The measurements of **(A)** weight and **(B)** white blood cell (WBC) count in sheep with fracture-related infection receiving local phage therapy (PT) combined with intravenous vancomycin, intravenous PT combined with intravenous vancomycin and intravenous vancomycin only. Weights were normalized to the preoperative weights at the primary surgery and set at 100%. Data points represented individual measurements, with horizontal lines indicating the mean values and statistical analysis was performed using a two-way ANOVA followed by *post hoc* Tukey’s multiple comparisons test. The detection limit of the assay used to quantify bacteria was 10 CFU/mL.

Supplementary Figure 4 Bacterial load in different tissue locations: proximal bone, distal bone, and bone marrow for each animal. The treatments compared were local PT combined with intravenous vancomycin, intravenous PT combined with intravenous vancomycin, and vancomycin alone. Data represent the mean ± standard deviation, and statistical significance was determined using a Kruskal-Wallis test followed by Tukey’s posttest. CFU, colony-forming units.
