# Supplementary material for: Evaluating the safety, pharmacokinetics and efficacy of phage therapy in treating fracture-related infections with multidrug-resistant Staphylococcus aureus: intravenous versus local application in sheep
Source: Front Cell Infect Microbiol. 2025 Apr 4;15:1547250. doi: 10.3389/fcimb.2025.1547250 (PMC12006137; doi:10.3389/fcimb.2025.1547250)
Supplement: Supplementary Figure 1 — The placement of the probe and the collection tube for the animals that received local administration. The left side is phase 1, animal without any osteotomy or fracture-related infection, and the right side is phase 2, where the animals received an osteotomy, plate ad screw fixation and fracture-related infection. The animals that received systemic administration (i.e. no PT) did not receive any tube for phage application. [file Image1.pdf]

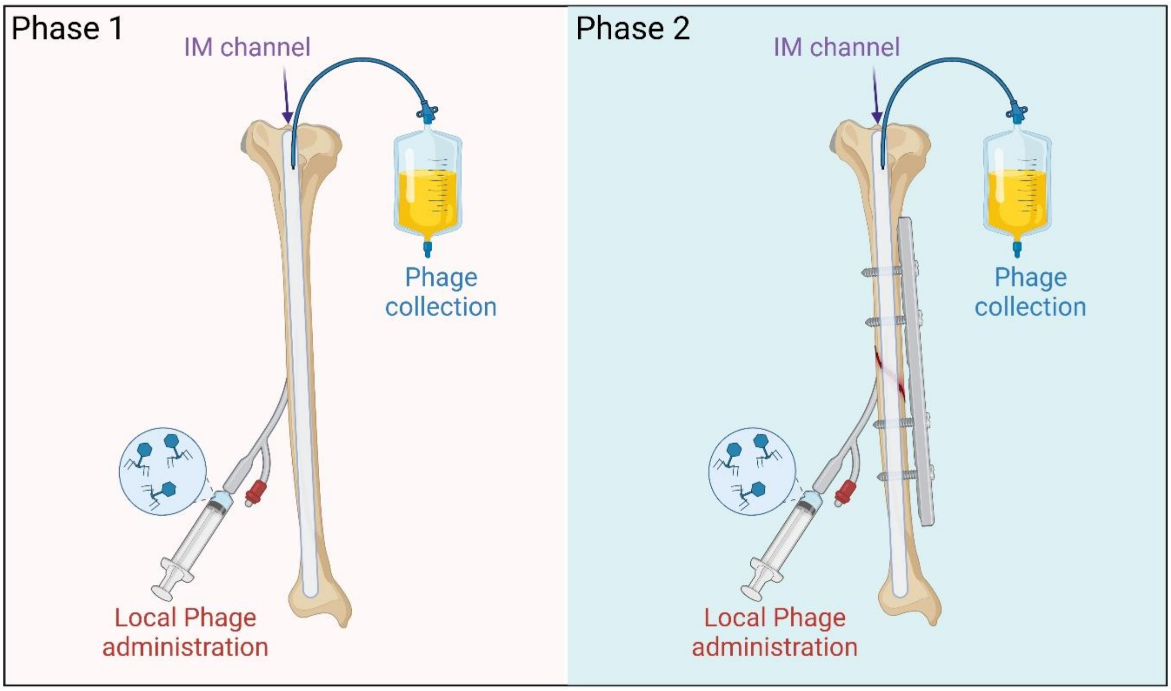

Figure S1. The placement of the probe and the collection tube for the animals that received local administration. The left side is phase 1, animal without any osteotomy or fracture-related infection, and the right side is phase 2, where the animals received an osteotomy,plate ad screw fixation and fracture-related infection. The animals that received systemic administration (i.e. no PT) did not receive any tube for phage application.

(A)

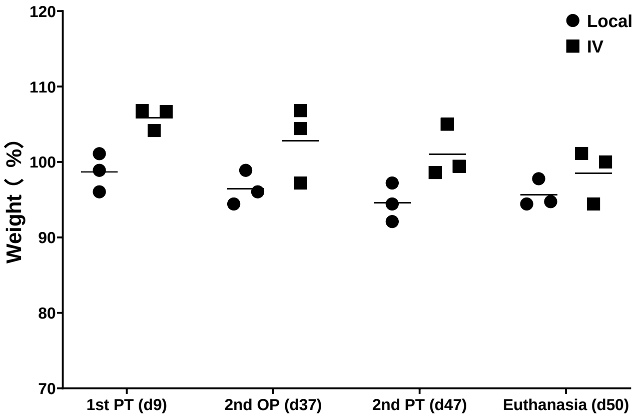

(B)

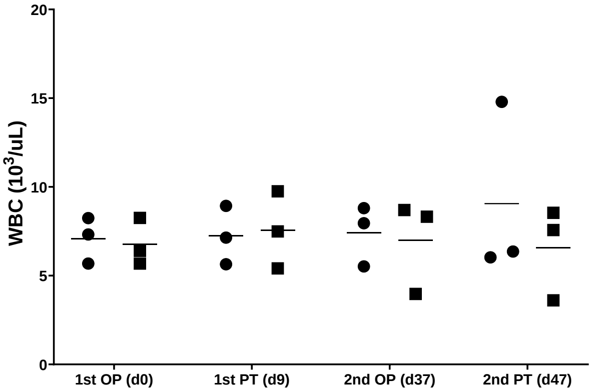

Figure S2. The measurements of (A) weight and (B) white blood cell (WBC) count in sheep receiving phage administration locally and intravenously. Weights were normalized to the preoperative weights at the primary surgery and set at 100%. 1st OP: The beginning of PT. 1st PT: D9 is the timepoint when PT of the first round has been administered for 10 days; 2nd OP: 28 days after the end of PT, the second operation started at Day 37; 2nd PT: D47 was the timepoint when PT of the second round has been administered for 10 days. Data points represented individual measurements, with horizontal lines indicating the mean values and statistical significance was determined using a student's t-test.

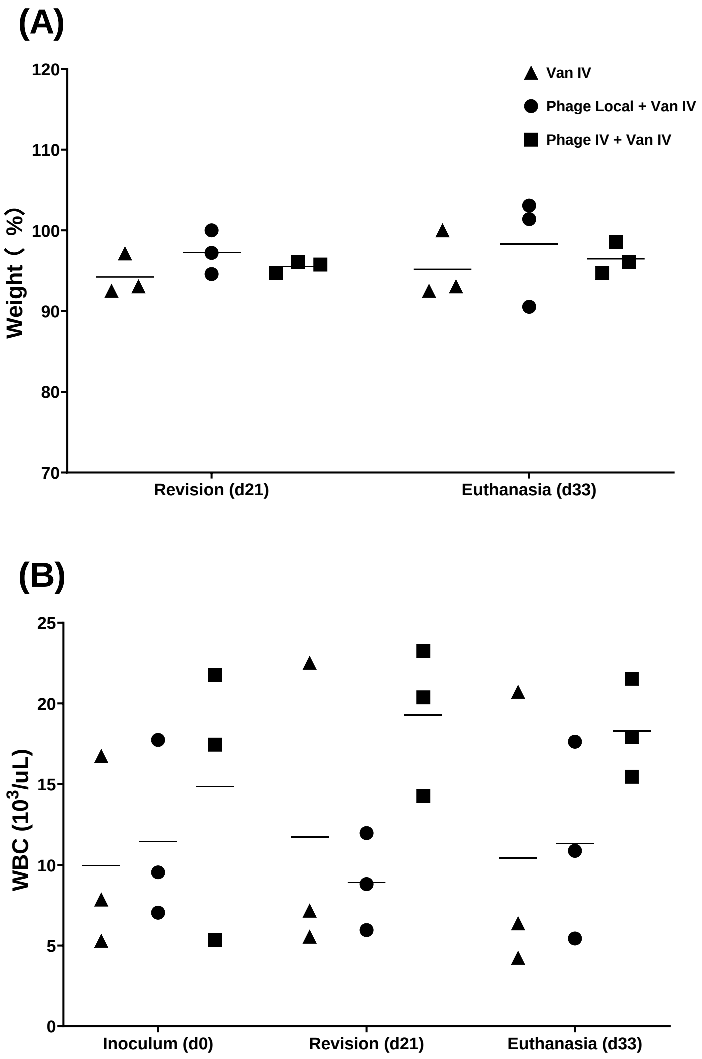

Figure S3. The measurements of (A) weight and (B) white blood cell (WBC) count in sheep with fracture-related infection receiving local phage therapy (PT) combined with intravenous vancomycin, intravenous PT combined with intravenous vancomycin and intravenous vancomycin only. Weights were normalized to the preoperative weights at the primary surgery and set at 100%. Data points represented individual measurements, with horizontal lines indicating the mean values and statistical analysis was performed using a two-way ANOVA followed by post hoc Tukey's multiple comparisons test. The detection limit of the assay used to quantify bacteria was 10 CFU/mL.

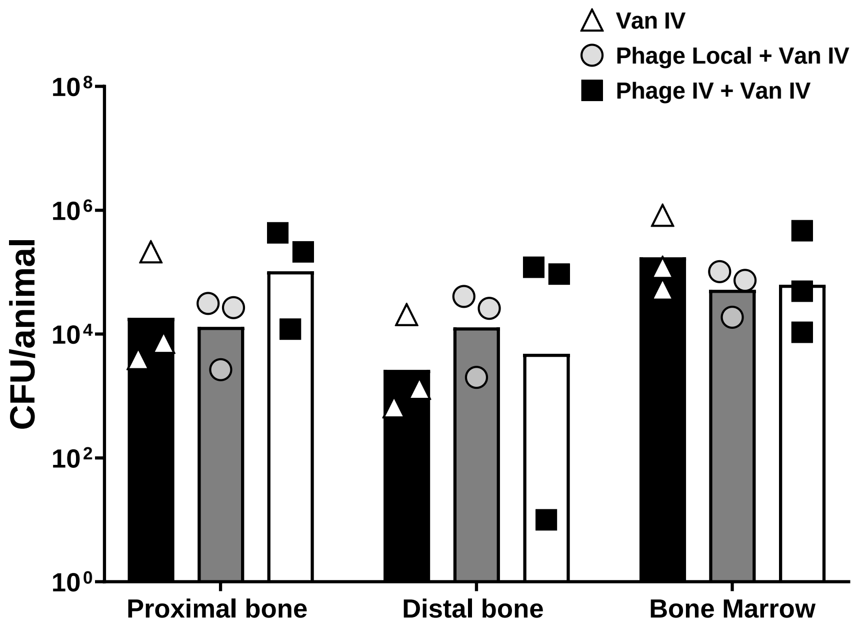

Figure S4. Bacterial load in different tissue locations: proximal bone, distal bone, and bone marrow for each animal. The treatments compared were local PT combined with intravenous vancomycin, intravenous PT combined with intravenous vancomycin, and vancomycin alone. Data represent the mean ± standard deviation, and statistical significance was determined using a Kruskal-Wallis test followed by Tukey's posttest. CFU, colony-forming units.
